# Severe Babesiosis With Lyme Disease and Anaplasma Phagocytophilum Coinfection in a Dialysis-Dependent Patient Without Rash or Organomegaly

**DOI:** 10.7759/cureus.105088

**Published:** 2026-03-12

**Authors:** Michelle Raja, Sona Sharma

**Affiliations:** 1 Internal Medicine, New York Institute of Technology College of Osteopathic Medicine, New York, USA; 2 Internal Medicine, Icahn School of Medicine at Mount Sinai, Queens Hospital Center, New York, USA

**Keywords:** babesia microti, babesiosis, dialysis dependent, end-stage renal disease, hemolytic anemia, high parasitemia, ixodes borne infection, lyme coinfection, tick-borne disease, triple infection

## Abstract

Babesiosis is a tick-borne intraerythrocytic protozoal infection endemic to the northeastern United States. Coinfection with other *Ixodes*-transmitted pathogens may occur due to shared vector exposure, although confirmed triple infection remains uncommon. Severe babesiosis, defined by parasitemia ≥10% or evidence of organ dysfunction, may present atypically in immunocompromised individuals.

A 73-year-old man with end-stage renal disease on hemodialysis presented with two weeks of cyclic fevers, chills, and weakness after hiking in upstate New York. Physical examination revealed asterixis without rash, jaundice, or organomegaly. Laboratory evaluation demonstrated hemoglobin 6.9 g/dL, platelet count 124 K/µL, lactate dehydrogenase 709 U/L, and 13% parasitemia on peripheral smear. Polymerase chain reaction confirmed *Babesia microti*. Serologic testing revealed positive Lyme IgG and IgM antibodies and elevated *Anaplasma phagocytophilum* IgG titers. The patient was treated with azithromycin, atovaquone, and doxycycline, resulting in parasitemia clearance by hospital day 7 without exchange transfusion.

Severe babesiosis with *Ixodes*-borne coinfection may occur without classic findings such as rash or hepatosplenomegaly, particularly in dialysis-dependent patients. Persistent fever in endemic regions despite doxycycline therapy should prompt evaluation for babesiosis. Early recognition and individualized management may prevent invasive interventions.

## Introduction

Babesiosis is a malaria-like illness caused by intraerythrocytic protozoa of the genus *Babesia*, most commonly *Babesia microti*, in the United States. Transmission occurs through the bite of the* Ixodes scapularis* tick, which also transmits *Borrelia burgdorferi *and *Anaplasma phagocytophilum*. The disease is most commonly reported in the Northeastern United States and the Upper Midwest, although cases have also been reported in the Pacific Northwest [1,2]. Coinfection with Lyme disease or anaplasmosis occurs in up to 20%-50% of babesiosis cases; however, confirmed triple infection remains uncommon, reported in approximately 7.5% of babesiosis-positive cohorts [3]. Immunocompromised patients, including those with end-stage renal disease (ESRD), are at increased risk for severe babesiosis due to impaired immune clearance and baseline hematologic abnormalities [4]. We present a case of severe babesiosis with concurrent serologic evidence of Lyme disease and anaplasmosis in a dialysis-dependent patient presenting without rash or organomegaly.

## Case presentation

A 73-year-old man with ESRD receiving thrice-weekly hemodialysis presented with swelling over his left upper-extremity arteriovenous fistula. His medical history was notable for chronic hepatitis B, hypertension, type 2 diabetes mellitus, hyperlipidemia, benign prostatic hyperplasia, and chronic anemia. He reported two weeks of intermittent fevers, chills, weakness, tremors, and malaise following hiking trips in upstate New York. He recalled a tick bite with a transient rash but denied persistent dermatologic findings.

Vital signs were stable on admission. Physical examination revealed asterixis without rash, scleral icterus, lymphadenopathy, or hepatosplenomegaly.

Laboratory studies demonstrated leukocytosis of 12.8 K/µL, hemoglobin of 6.9 g/dL, platelet count of 124 K/µL, and blood urea nitrogen consistent with his ESRD baseline. Hemolysis markers included lactate dehydrogenase of 709 U/L, indirect bilirubin of 1.3 mg/dL, haptoglobin of <10 mg/dL, and a reticulocyte count of 6.2%. Additional liver function tests showed aspartate aminotransferase of 52 U/L, alanine aminotransferase of 48 U/L, and alkaline phosphatase of 132 U/L.

Peripheral blood smear revealed intraerythrocytic ring forms consistent with *Babesia microti*, with an estimated parasitemia of 13%. Maltese-cross tetrads were not observed, consistent with prior reports noting their infrequent appearance [5]. Repeat smear on hospital day 2 demonstrated parasitemia of 11.1%, and smears obtained on hospital day 7 were negative.

Polymerase chain reaction (PCR) performed on peripheral blood specimens confirmed *Babesia microti* infection. Lyme serologies were positive with elevated IgG and IgM titers, while Lyme PCR was negative. *Anaplasma phagocytophilum* IgG titers were elevated at 1:512 with negative IgM. Malaria antigen testing was negative. Computed tomography of the abdomen and pelvis demonstrated normal liver and spleen without organomegaly.

Treatment and outcome

Before the infectious disease consultation, the patient received a single empiric dose of artemether-lumefantrine for malaria. He was transfused one unit of packed red blood cells and initiated on azithromycin 500 mg orally once daily and atovaquone 750 mg orally twice daily for treatment of babesiosis, along with doxycycline 100 mg orally twice daily for coverage of potential tick-borne coinfection, including Lyme disease and anaplasmosis. The patient completed a 10-day course of azithromycin and atovaquone and was discharged on an extended 14-day course of doxycycline.

Serial monitoring demonstrated a progressive decline in parasitemia with clearance by hospital day 7. Lactate dehydrogenase decreased from 709 U/L to 581 U/L, platelet count increased from 124 K/µL to greater than 300 K/µL, and hemoglobin stabilized between 7 and 8 g/dL. Exchange transfusion was considered due to parasitemia exceeding 10%, but was deferred given clinical stability and laboratory improvement. He remained afebrile and tolerated outpatient hemodialysis without recurrence. Hematologic abnormalities and hemolysis markers are summarized in Table 1. 

**Table 1:** Initial hematologic and hemolysis laboratory findings in severe babesiosis

Laboratory test	Patient value	Reference range
White blood cell count	12.8 K/µL	4.0-11.0 K/µL
Hemoglobin	6.9 g/dL	13.5-17.5 g/dL
Platelet count	124 K/µL	150-450 K/µL
Lactate dehydrogenase (LDH)	709 U/L	140-280 U/L
Indirect bilirubin	1.3 mg/dL	0.1-1.0 mg/dL
Haptoglobin	<10 mg/dL	30-200 mg/dL
Reticulocyte count	6.2%	0.5%-2.5%
Parasitemia (peripheral smears)	13%	0%

## Discussion

This case demonstrates severe babesiosis with concurrent serologic evidence of *Borrelia burgdorferi* and *Anaplasma phagocytophilum* exposure in a dialysis-dependent patient presenting without rash, jaundice, or hepatosplenomegaly. Such atypical presentations are increasingly recognized in immunocompromised hosts [4]. A review of 139 hospitalized cases of babesiosis in New York State demonstrated that severe disease is more likely in older and immunocompromised patients, with parasitemia, anemia, and thrombocytopenia serving as important prognostic indicators [6].

Babesiosis may be misdiagnosed as malaria due to their shared intraerythrocytic parasitic infection and similar clinical manifestations, including fever, hemolytic anemia, and thrombocytopenia. However, the two diseases require different treatment regimens, making an accurate diagnosis essential.

ESRD may further predispose patients to severe babesiosis through immune dysregulation. Uremia is associated with impaired neutrophil chemotaxis and phagocytosis, reduced complement activity, altered T-cell function, and chronic systemic inflammation. Additionally, hemodialysis may contribute to immune exhaustion and diminished pathogen clearance. In the setting of baseline anemia and reduced erythrocyte reserve, these factors may facilitate higher parasitemia levels and more pronounced hemolysis than immunocompetent hosts, consistent with observations that immunocompromised patients are at increased risk for severe disease [4].

Although dual *Ixodes*-borne coinfection is well documented, confirmed triple infection remains uncommon and may complicate diagnosis [3]. In this case, positive *Borrelia burgdorferi* IgM and IgG antibodies with negative PCR, along with elevated *Anaplasma* IgG titers in the absence of IgM, may represent prior exposure rather than acute infection, as isolated elevated IgG titers without IgM or PCR confirmation cannot reliably distinguish past infection from active disease. However, treatment with doxycycline was pursued, given endemic exposure risk and overlapping clinical features.

Current Infectious Diseases Society of America guidelines recommend individualized consideration of exchange transfusion for parasitemia ≥10%, particularly in the presence of end-organ dysfunction [3]. Emerging data support conservative management in clinically stable patients demonstrating rapid parasitemia reduction [3].

The incidence of babesiosis, Lyme disease, and anaplasmosis continues to increase in the northeastern United States, paralleling the expansion of tick habitats and prolonged transmission seasons [7]. Clinicians should maintain a high index of suspicion for babesiosis in patients from endemic regions presenting with persistent fever despite doxycycline therapy. Recent reports have also described severe and occasionally fatal coinfection with *Babesia microti* and *Borrelia burgdorferi*, highlighting the diagnostic and therapeutic challenges associated with overlapping tick-borne infections.

Several previously reported cases of tick-borne coinfection describe similar presentations with fever, hemolysis, and thrombocytopenia in patients from endemic regions. However, the present case is notable for the combination of severe parasitemia (>10%) in a dialysis-dependent patient and the absence of classic clinical findings such as rash, jaundice, or hepatosplenomegaly, which may delay recognition of babesiosis in immunocompromised hosts. This highlights the importance of maintaining clinical suspicion for babesiosis even when typical features are absent.

This case highlights several key clinical lessons: severe babesiosis may present without classic findings such as rash or hepatosplenomegaly; coinfection with other *Ixodes*-borne pathogens may complicate diagnosis, and immunocompromised patients, including those receiving hemodialysis, may develop severe disease even in the absence of typical clinical features. Early recognition and appropriate antimicrobial therapy are essential to prevent complications and unnecessary invasive interventions.

## Conclusions

Severe babesiosis with *Ixodes*-borne coinfection may present without classic findings such as rash or organomegaly, particularly in dialysis-dependent patients. Immune dysregulation and baseline hematologic abnormalities associated with ESRD may contribute to higher parasitemia and more pronounced hemolysis. Clinicians practicing in endemic regions should maintain a high index of suspicion in patients with persistent fever despite doxycycline therapy, as early recognition and appropriate antimicrobial therapy may prevent complications and reduce the need for invasive interventions such as exchange transfusion.
